# Serum Uric Acid and Progression of Kidney Disease: A Longitudinal Analysis and Mini-Review

**DOI:** 10.1371/journal.pone.0170393

**Published:** 2017-01-20

**Authors:** Ching-Wei Tsai, Shih-Yi Lin, Chin-Chi Kuo, Chiu-Ching Huang

**Affiliations:** 1 Division of Nephrology and Kidney Institute, Department of Internal Medicine, China Medical University Hospital, Taichung, Taiwan; 2 School of Medicine, College of Medicine, China Medical University, Taichung, Taiwan; 3 Big Data Center, China Medical University Hospital, Taichung, Taiwan; The University of Tokyo, JAPAN

## Abstract

**Background:**

Increasing evidence supports the association between hyperuricemia and incident chronic kidney disease (CKD); however, there are conflicting data regarding the role of hyperuricemia in the progression of CKD. This study retrospectively assessed the longitudinal association between uric acid (UA) level and CKD progression in a Chinese population lived in Taiwan.

**Methods:**

Patients with physician diagnosis of hyperuricemia or receiving urate-lowering therapy between 2003 and 2005 were identified in the electronic medical records (EMR) of a tertiary medical center and were followed up until December 31, 2011. Patients were divided into four UA categories at the cut-off 6, 8, and 10 mg/dL. CKD progression was estimated by the change of estimated glomerular filtration rate (eGFR) in the linear mixed models. Kidney failure was defined as an eGFR less than 15 mL/min/1.73 m^2^ or requiring renal replacement therapy.

**Results:**

A total of 739 patients were analyzed. In the full-adjusted model, patients with a baseline UA level ≥6 mg/dL had greater decline in eGFR ((*β* = -9.6, 95% CI -16.1, -3.1), comparing to those with a UA level less than 6 mg/dL. When stratifying patients into four UA categories, all three hyperuricemia categories (UA6-8, 8–10, ≥10 mg/dL) associated with a greater decline in eGFR over the follow-up period with an increasing dose-response, comparing to the lowest UA category. The risk of progression to renal failure increased 7% (hazard ratio 1.07, 95% CI 1.00, 1.14) for each 1mg/dL increase in baseline UA level. The influences of hyperuricemia on eGFR decline and the risk of kidney failure were more prominent in patients without proteinuria than those with proteinuria.

**Conclusion:**

Our study showed a higher uric acid level is associated with a significant rapid decline in eGFR and a higher risk of kidney failure, particularly in patients without proteinuria. Our findings suggest hyperuricemia is a potential modifiable factor of CKD progression.

## Introduction

Chronic kidney disease (CKD) is a global health care burden [1]. Identification of modifiable risk factors, such as hyperglycemia and hypertension, and implantation efforts to control these factors are imperative for CKD prevention. An elevated uric acid (UA) level is commonly observed in CKD patients; however, whether it is simply a biomarker of impaired kidney function or has a true pathogenic role in kidney function remains inconclusive [2, 3]. In experimental rat models, hyperuricemia-induced kidney injury including afferent arteriolopathy, glomerulosclerosis, and tubulointerstitial fibrosis [4–6] could be reversed by urate-lowering agents [7, 8].

As uric acid is primarily excreted by the kidneys, it is difficult to evaluate the causal influence of uric acid on the progression of CKD in epidemiological research [3]. Although a recent meta-analysis found that elevated serum UA levels were associated with incident CKD [9], the role of UA in CKD progression is still debating. For instance, results from a large cohort of the Swedish Renal Registry showed neither the rate of estimated glomerular filtration rate (eGFR) decline nor rapid progression to end stage renal disease (ESRD) was associated with serum UA levels in patients with CKD stage 3 to 5 [10]. This finding is concordant with some recent observational studies of patients with a wide range of renal function at baseline in the U.S. [11], Taiwan [12], and Europe (Germany, Austria, south Tyrol, and Netherlands)[13, 14]. However, large heterogeneity in the definitions of CKD progression and analytic methods among these studies precluded a firm conclusion. A similar controversy surrounds the role of urate-lowering agents in retarding CKD progression. While several studies supported the benefit of urate-lowering therapy in delaying the progression of CKD[15, 16], a recent meta-analysis of randomized trials did not support the beneficial effect of urate-lowering therapy on renal outcome [17]. The discrepancy may be related to the relatively small sample size of the included trials [17–20].

The current study aimed to contribute evidence from a longitudinal study to this ongoing debate about the role of serum UA level on CKD progression in a Chinese population-based sample from Taiwan. We also summarized published evidence about the effect of serum UA level on CKD progression.

## Materials and Methods

### Ethics statement

This study was approved by the Institutional Review Board of China Medical University Hospital (CMUH). Informed consent was not obtained from the study participants because the data was analyzed anonymously and was in accordance with Institutional Review Board guidelines. The Institutional Review Board has verified the anonymity of data analysis performed in this study.

### Study population

We conducted a retrospective cohort study at a tertiary medical center in Taiwan, which has adopted electronic medical records (EMR) since 2001. We consecutively selected patients who visited CMUH between 2003 and 2005 and have been diagnosed hyperuricemia [defined by uric acid (UA) greater than 7 mg/dL or by International Classification of Diseases, Ninth Revision, Clinical Modification (ICD-9-CM) codes: hyperuricemia (790.6)] [21] and/or receiving urate-lowering therapy for more than 1 months and were followed up till December 31, 2011 (S1 Fig). The timing of diagnosis of hyperuricemia and receiving urate-lowering therapy could early before 2003. The major urate-lowering therapy between 2003 to 2005 was allopurinol, while a few patients received sulfinpyrazone. The timing of diagnosis of hyperuricemia and receiving urate-lowering therapy could early before 2003. Baseline demographic information including age, sex, comorbidity, blood pressure and history of medication (e.g., allopurinol, benzbromarone, angiotensin converting enzyme inhibitors (ACEIs), angiotensin II receptor blockers (ARBs) were collected at the first outpatient visit that the individual met the diagnostic criteria mentioned above during the study period. Serial levels of serum creatinine were measured at follow-up visits. To specifically address the renal function trajectory, only patients with at least three available serum creatinine measurements between 2003 and 2011 were analyzed. Patients have been treated with dialysis or have had a kidney transplant at baseline or within 30 days after selection were excluded. Finally, a total of 739 patients were included in this study.

### Clinical and laboratory data

The eGFR was estimated using the abbreviated Modification of Diet in Renal Disease (MDRD) equation (eGFR = 175 × creatinine^-1.154^ × age^-0.203^ × 1.212 [if black] × 0.742 [if female]) [22]. The baseline eGFR and CKD stage were based on the first available eGFR. Patients were divided into five groups according to the baseline eGFR using the cut-off values: ≥ 90, 60–89.9, 30–59.9, 15–29.9, and <15 ml/min per 1.73 m^2^ [23]. Daily protein loss was quantified by a urine protein-creatinine ratio (UPCR) or an albumin-creatinine ratio (ACR) from a random spot urine sample. The diagnosis of proteinuria was confirmed in at least two of three consecutive urine examinations showing a urine dipstick of 1+ or greater, a UPCR > 150 mg/g creatinine, or an ACR > 300 mg/g creatinine [23]. Diabetes was defined as physician-reported diagnosis, active use of anti-diabetic agents, or having a fasting glucose level of 126 mg/dL or greater, a random glucose level of 200 mg/dL or greater, or a hemoglobin A1c level of 6.5% or greater. Hypertension was defined as systolic pressure ≥140 mmHg, diastolic pressure ≥90 mmHg, self-reported hypertension, or the use of an antihypertensive medication. Brachial blood pressure (BP) was measured by an automated oscillometric BP device at clinic visits after sitting for at least 5 minutes (Omran HBP-9020 Blood Pressure Monitor; Kyoto, Japen). History of cardio-vascular disease (CVD) was defined based on any documented diagnosis of coronary artery disease, myocardial infarction, stroke, or heart failure. CKD progression was evaluated by two different models: (1) renal function trajectory approximated by the changing slope of eGFR using linear mixed models and (2) incident kidney failure (defined as patient’s eGFR declining to <15 mL/min/1.73 m^2^ and/or receiving chronic dialysis therapy) using cox proportional hazard model.

### Statistical analyses

Baseline characteristics are presented for the total study population stratified by serum UA level (UA<6, 6–8, 8–10, and ≥10 mg/dL) with UA<6 as the reference group, based on the saturation point for monosodium urate [24–26] and the therapeutic goal of urate lowering therapy in the current guidelines[27, 28]. The cut-offs values to categorize serum uric acid into quartiles were based on clinical experience [29]. The categorization of uric acid level could help evaluate the dose-response relationship between CKD progression and serum uric acid. Continuous variables were presented as mean values with standard deviation (SD), and categorical variables were expressed as frequencies and percentages. Statistical differences among the UA strata were evaluated by chi-square test and one-way ANOVA for categorical and continuous variables, respectively.

The primary outcome was individual eGFR trajectory during outpatient follow-up. As repeated eGFR measurements are correlated within each patient, linear mixed model (LMM) with random intercept and random slope was used to evaluate the longitudinal change in eGFR for each UA category at baseline. Based on Akaike Information Criterion (AIC), the unstructured covariance matrix was selected to handle the with-person correlation and improve the data fitting. For incident kidney failure, multiple Cox proportional-hazard regression model was performed using age as the time scale. We treated age at each examination as a time varying co-variable. To handle left-truncation induced at the time of selection and appropriately align risk sets on the age scale, the late entry method was conducted using age at baseline as the individual entry time. Multivariable modeling was performed after adjusting for potential confounders: age at examination, sex, body mass index (BMI), presence of cardiovascular disease (CVD), systolic blood pressure, diabetes mellitus (yes vs. no), proteinuria (yes vs. no), baseline serum creatinine, ACEI/ARB use (yes vs. no) and allopurinol use (yes vs. no). A priori exploratory subgroup analysis stratified by proteinuria status was also performed using similar modeling process. The interactions between uric acid and proteinuria were tested in multivariable models for serial eGFR change and risk of ESRD. All analyses were conducted using Stata, version 12 (Stata, College Station, TX). The 2-sided statistical significance level was set at α = 0.05.

## Results

### Patient characteristics

A total of 739 patients were analyzed with a mean follow-up of 4.25 years.

**Fig 1:**
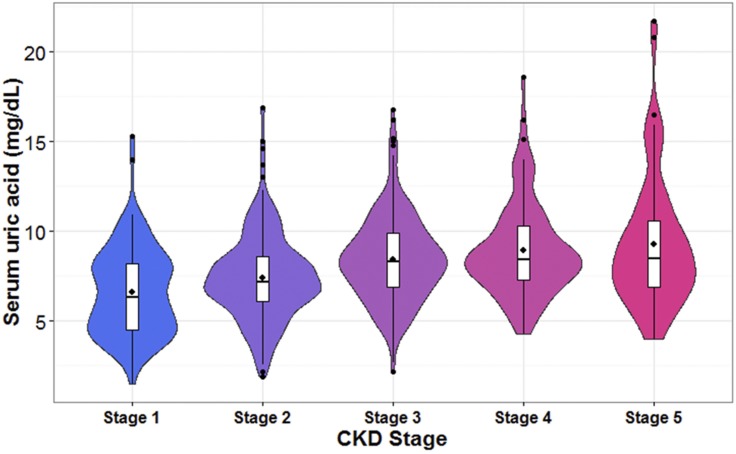
Violin plot of the distribution of baseline serum uric acid levels by CKD stages. The violin is a mirrored density plot with a boxplot of the baseline uric acid concentrations inside. Black dots represented the group mean and outliers in each CKD category.

**Fig 2:**
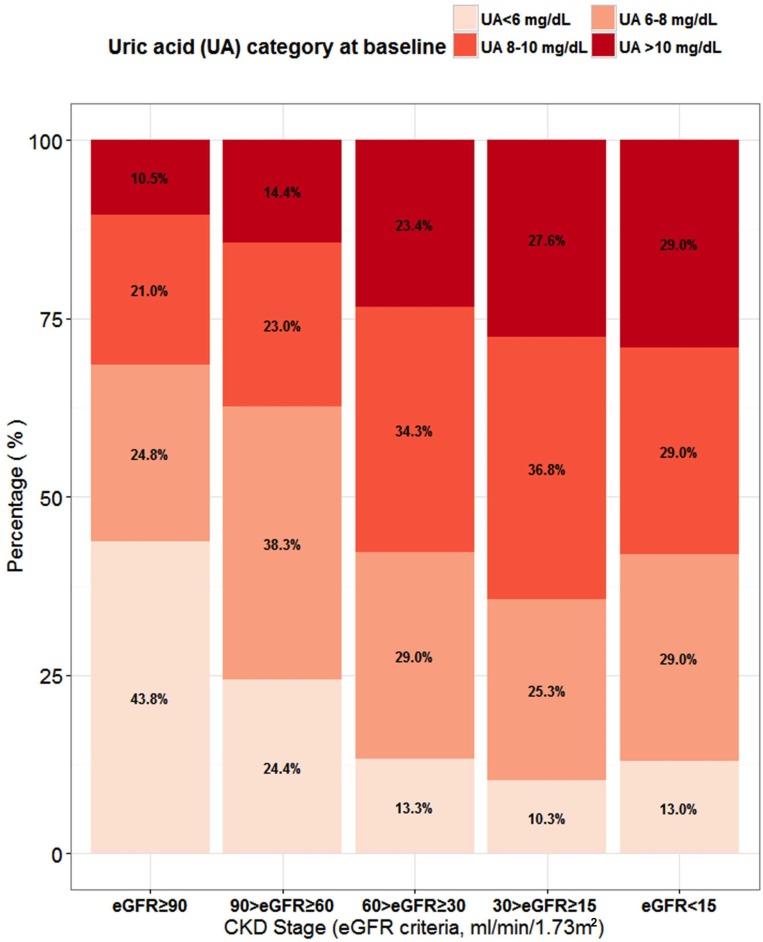
The distribution of the proportion of baseline hyperuricemic status (by cut-off values of 6, 8, and 10mg/dL) across CKD stages.

Baseline characteristics across baseline UA categories are summarized in Table 1. There was no significant difference at baseline age, lipid profile and the prevalence of hypertension and CVD (Table 1). Patients with UA<6 mg/dL had higher prevalence of diabetes compared to those with UA ≥ 6 mg/dL. There was an increasing trend in the mean serum creatinine concentration with a corresponding decreasing trend in eGFR across increasing UA categories (*p*-trend <0.001 and <0.001, respectively, Table 1). Patients with higher baseline UA levels were also more likely to receive allopurinol (*p* -trend <0.001, Table 1). When we stratified the patients by baseline CKD stage, an increasing trend in both mean UA levels and the proportion of patients with an UA level greater than 10 mg/dL was observed with increasing CKD stage (Figs 1 and 2).

**Table 1:** Characteristics of hyperuricemic patients stratified by baseline serum uric acid categories*.

Serum uric acid level	<6	6–8	8–10	>10	*p*-trend
(mg/dL)	N = 153	N = 228	N = 212	N = 146	
***Demographics***					
Age (years)	63.0 (1.2)	63.6 (1.0)	62.7 (1.0)	62.8 (1.2)	1.0
Male (%)	95(62.1%)	132 (60.5%)	122 (62.3%)	97 (67.8%)	0.5
Smoking (%)	59 (38.6%)	80 (35.1%)	79 (37.2%)	64 (43.8%)	0.4
***Comorbidity***					
Hypertension (%)	88 (57.5%)	145 (65.6%)	138 (69.8%)	97 (67.8%)	0.09
Cardiovascular disease (%)	26 (17.0%)	53 (23.3%)	48 (22.6%)	34 (23.3%)	0.5
Diabetes (%)	72 (47.1%)	95 (41.7%)	75 (35.6%)	53 (36.3%)	0.1
***Baseline clinical characteristics***					
Systolic blood pressure (mmHg)	136.3 (2.0)	137.7 (1.6)	138.4 (1.7)	139.7(2.1)	0.3
Diastolic blood pressure (mmHg)	75.4 (2.2)	77.1 (1.8)	81.7 (1.9)	79.4(2.3)	0.1
Body mass index (kg/m^2^)	24.0 (0.4)	25.1 (0.3)	25.1 (0.3)	25.3 (0.4)	0.03
Serum total cholesterol (mg/dL)	191.9(5.7)	197.7 (4.7)	197.2 (5.0)	203.0 (6.0)	0.2
Serum triglyceride (mg/dL)	221.8 (22.6)	187.4 (18.8)	183.9(19.9)	176.2 (24.1)	0.2
Serum creatinine (mg/dL)	1.58 (0.18)	1.80 (0.15)	2.13 (0.16)	2.51 (0.19)	<0.001
Proteinuria (Yes vs. No) (%)	61 (43.3%)	100 (47.6%)	92 (45.5%)	63 (49.2%)	0.8
eGFR (ml/min/1.73m^2^)	74.7 (2.5)	57.9 (2.1)	52.6 (2.2)	46.3 (2.7)	<0.001
***Baseline medication***					
Allopurinol (%)	32 (20.9%)	66 (29.0%)	87 (41.0%)	79 (54.1%)	<0.001
ACEI/ARB (%)	70 (45.8%)	123 (54.0%)	127 (59.9%)	84 (57.5%)	0.05

* Characteristics of CKD patients are given as percentage for each categorical variable (e.g., hypertension) or arithmetic mean (standard error) (e.g., age) for each continuous variable.

Abbreviations: ACEI/ARB, angiotensin converting enzyme inhibitor/ AT1 receptor antagonist; eGFR, estimated glomerular filtration rate.

### The association among uric acid, and the decline in renal function

Longitudinal analyses showed patients with hyperuricemia (a baseline UA level ≥ 6 mg/dL) (*β* = -9.6, 95% CI -16.1, -3.1), had a greater decline in eGFR during follow-up, comparing to those with UA level < 6 mg/dL. For every one mg/dl increase in baseline UA level, the decline in eGFR was significantly greater during follow-up (*β* = -1.6, 95% CI -2.2, -1.0, Table 2, Model 4). In the fully-adjusted model, the slopes of eGFR decline were significantly steeper with increasing categories of hyperuricemia, comparing to those with UA <6 mg/dL (*β* = -11.2, -12.6, -13.1 for UA level 6–8, 8–10, ≥ 10 mg/dL, respectively, *p*-trend<0.001) (Table 2, Model 4). As proteinuria and a higher baseline serum creatinine concentration were also strongly associated with a greater eGFR decline during follow-up (proteinuria: *β* = -9.1, p<0.001; baseline serum creatinine: *β* = -7.4, p<0.001)(S1 Table), so did the Allopurinol use (*β* = -9.8, p<0.001) even after adjusted for baseline UA level (S1 Table). In interaction analysis between proteinuria and UA, the influence of hyperuricemia on renal function decline was greater in subjects without proteinuria (*β* = -2.3, 95% CI -3.2, -1.5) than in those with proteinuria (*β* = -1.4, 95% CI -2.4, -0.4) (*P* value for interaction term between uric acid level and proteinuria status are <0.01 in model 3 and 4) (Table 2, subgroup analysis, model 3).

**Table 2:** Change in eGFR for each 1 mg/dL increase in serum uric acid level and across uric acid (UA) categories with subgroup analysis stratified by proteinuria status.

	Model 1	Model 2	Model 3	Model 4
Change in eGFR	β (95% CI)	β (95% CI)	β (95% CI)	β (95% CI)
Serum uric acid level (mg/dL)	-3.3 (-4.0, -2.5)	-3.3 (-4.1, -2.6)	-2.9 (-3.7, -2.2)	-1.6 (-2.2, -1.0)
Uric acid category				
<6 mg/dL	Reference	Reference	Reference	Reference
6–8 mg/dL	-14.5 (-20.4, -8.5)	-14.1 (-19.8, -8.4)	-12.2 (-17.0, -7.4)	-11.2 (-15.8, -6.6)
8–10 mg/dL	-19.8 (-25.9, -13.8)	-19.4(-25.2, -13.6)	-15.1 (-20.0, -10.2)	-12.6 (-17.4, -7.8)
>10 mg/dL	-24.6 (-31.3, -17.9)	-25.3 (-31.7, -18.9)	-17.0 (-22.5, -11.5)	-13.1 (-18.5, -7.6)
*p*-trend	<0.001	<0.001	<0.001	<0.001
**Subgroup analysis stratified by proteinuria status**				
Uric acid level (mg/dL)				
No Proteinuria	-3.9 (-5.0,-2.9)	-3.7 (-4.7,-2.7)	-2.3 (-3.2,-1.5)	-1.9 (-2.7, -1.0)
Proteinuria	-2.5 (-3.8,-1.3)	-2.3 (-3.5,-1.1)	-1.4 (-2.4,-0.4)	-0.9 (-1.8, 0.1)
Without proteinuria				
Uric acid category				
<6 mg/dL	Reference	Reference	Reference	Reference
6–8 mg/dL	-15.8 (-23.5, -8.1)	-15.5 (-22.6, -8.3)	-11.7 (-17.7, -5.7)	-10.2 (-16.1, -4.4)
8–10 mg/dL	-25.7 (-33.4, -17.9)	-23.7 (-31.0, -16.5)	-17.0 (-23.2, -10.7)	-14.4 (-20.5, -8.2)
>10 mg/dL	-27.0 (-36.0, -18.0)	-26.8 (-35.1, -18.5)	-18.9 (-26.0, -11.9)	-15.4 (-22.4, -8.3)
*p*-trend	<0.001	<0.001	<0.001	<0.001
With proteinruia				
Uric acid category				
<6 mg/dL	Reference	Reference	Reference	Reference
6–8 mg/dL	-11.1 (-20.8, -1.4)	-7.9 (-17.4, 1.6)	-10.4 (-17.9, -2.9)	-9.7 (-16.9, -2.6)
8–10 mg/dL	-12.6 (-22.5, -2.7)	-10.0 (-19.8, -0.3)	-9.1 (-16.8, -1.4)	-6.5 (-13.9, 1.0)
> 10 mg/dL	-19.0 (-29.7, -8.2)	-17.2 (-27.7, -6.8)	-12.0 (-20.3, -3.7)	-7.2 (-15.4, 0.9)
*p*-trend	<0.001	<0.001	0.01	0.21

Model 1: Univariable analysis; Model 2: Adjusted for age, sex, and body mass index; Model 3: Further adjusted for hypertension, diabetes, cardiovascular disease, baseline creatinine, proteinuria (yes/no); Model 4: Further adjusted for allopurinol, angiotensin-converting-enzyme inhibitor/angiotensin receptor blockers.

### The association between uric acid and risk of progression to kidney failure

A total of 144 patients developed kidney failure during the follow-up period. Using multivariable Cox proportional hazards models, the risk of progression to kidney failure increased by 7% (adjusted hazard ratio: 1.07, 95% CI 1.00, 1.14, p = 0.039) for every 1 mg/dL increase in baseline UA level (Table 3). ACEI/ARB use was associated with a lower risk of kidney failure (adjusted HR: 0.6, 95% CI 0.40, 0.92, S2 Table) comparing to those did not use. In contrast, allopurinol was not associated with the risk of kidney failure (adjusted HR 0.75, 95% CI 0.50, 1.13, S2 Table). When performing interaction analysis between proteinuria and UA, the influence of hyperuricemia on the risk of kidney failure was greater in subjects without proteinuria (HR 1.27, 95% CI 1.10, 1.46) than in those with proteinuria (HR 1.07, 95% CI 0.99, 1.17) (Table 3, Model 4) (*P* value for interaction term between uric acid level and proteinuria status are <0.05 in model 3 and 4).

**Table 3:** The risk of CKD progression to kidney failure (eGFR <15 ml/min) for each 1 mg/dL increase in uric acid level.

	Model 1	Model 2	Model 3	Model 4
	HR (95% CI)	HR (95% CI)	HR (95% CI)	HR (95% CI)
Uric acid level (mg/dl)				
All	1.14 (1.07, 1.21)	1.14 (1.08, 1.21)	1.07 (1.00, 1.14)	1.07 (1.00, 1.14)
Proteinuria	1.09 (1.01, 1.17)	1.12 (1.03, 1.21)	1.07 (0.98, 1.16)	1.07 (0.99, 1.17)
No Proteinuria	1.29 (1.14, 1.45)	1.30 (1.15, 1.47)	1.22 (1.07, 1.40)	1.27 (1.10, 1.46)
P value for interaction between proteinuria and uric acid			0.020	0.024

Model 1: Univariable analysis; Model 2: Adjusted for age, sex, and body mass index; Model 3: Further adjusted for hypertension, diabetes, cardiovascular disease, baseline creatinine, proteinuria (yes/no); Model 4: Further adjusted for allopurinol, angiotensin-converting-enzyme inhibitor/angiotensin receptor blockers.

## Discussion

Our study supports that hyperuricemia is associated with a greater decline in renal function and a higher risk of progressing to kidney failure. The influences of hyperuricemia on renal function decline and the risk of kidney failure are greater in patients without proteinuria than those with proteinuria. We also found that patients treated with allopurinol had a greater eGFR decline, though the risk of progression to kidney failure did not significantly increase, comparing to those who did not use urate-lowering agents.

Evidence for the role of hyperuricemia in the CKD progression is controversial[30]. Although some studies showed no association between hyperuricemia and the progression of CKD [10–14], other studies found hyperuricemia may hasten the failing of renal function measured by eGFR or increase the risk of developing ESRD(Table 4) [31–42]. Among 63,758 subjects with baseline eGFR ≥ 60 ml/min/1.73 m^2^, the subjects with hyperuricemia had an average annual decline of eGFR of 2.5 ± 9.5 mL/min/1.73 m^2^, that was almost twice faster than those of patients with normal uric acid levels [31]. Bellomo *et al*. followed 824 healthy people for about 5 years and found that UA was an independent risk factor for a significant loss of renal function (defined as a decrease in eGFR ≥10 ml/min/1.73m^2^ over 5 years)[32]. Chonchol *et al*. also found the adjusted odds ratio for rapid progression of kidney function (defined as a decrease in eGFR ≥ 3 mL/min/1.73 m^2^ / year) was 1.14 (95% CI, 1.04 to 1.24) for each 1 mg/dL increase in UA level in 5,808 elders in the Cardiovascular Health Study [34]. In our study, the effect of hyperuricemia on the decline of eGFR was more prominent among patients without proteinuria compared to those with proteinuria. It is possible that in patients with proteinuria, chronic glomerulonephritis is the main underlying renal insult in which proteinuria is the major predictor for CKD progression and the influence of uric acid is less essential. For those without proteinuria, the underlying etiologies are more like to be tubulointerstitial nephritis, including urate nephropathy, hyperuricemia stands out as a strong prognostic factor for CKD progression. However, this aspect needs to be investigated in future research.

**Table 4:** Studies of serum uric acid level in the progression of kidney function and the development of kidney failure.

1^st^ author^#^, year	Sample size	Study population	Outcome definition of CKD progression	Risk estimation (95% CI)	Adjustment Factors
		- Characteristics			
		- Mean study duration (years)			
**Studies showed no association**					
Strum^13^	177	- Not-diabetic CKD stage 1–5	SCr doubling and/or ESRD	HR: 1.03 (0.85, 1.26)	eGFR, age, sex, proteinuria
2008				(per 1 mg/dL↑ in UA)	
		- 47 months			
Madero^11^	840	- CKD	ESRD	HR 1.20 (0.95, 1.51)	Age, sex
2009				[Highest vs. lowest tertile UA]	
	
Liu^12^	3,303	CKD stages 3–5	1) Rapid renal progression (the slope of eGFR was **>**6 ml/min/1.73 m2/y)	1) HR 1.30 (0.98, 1.73)	eGFR, Age, sex, CVD, MAP, BMI, HbA1C, T-CHO, smoker, log-CRP, proteinuria, Alb, Hb, HCO3, Ca, P, ACEI, ARB, diuretics, gout
				[Highest vs. lowest quartile UA]	
2012		- 2.8 years	2) Time to RRT	2) HR 0.96 (0.79, 1.16)	
				[Highest vs. lowest quartile UA]	
Nacak^14^	131	- CKD stages 4–5	1) The change in the rate of decline in eGFR	1) β = −0.14 (−0.70, 0.42)	Age, sex, comorbidity, diet, BMI, BP, lipid, proteinuria, diuretics, allopurinol
				[per 1 mg/dL↑ in UA] (LMM)	
2014		- 14.9 months	2) Time to RRT	2) HR 1.26 (1.06, 1.49)	
				[per 1 mg/dL↑ in UA]	
Nacak^10^	2466	CKD stages 3–5	1) Annual decline of eGFR	1) β = 0.09 (**−**0.01, 0.19)	Age, sex, PRD, BMI, MAP, protein-restricted diet, diuretics, statin, UA-lowering medication, DM, arrhythmia, dementia, CVD, IHD, HTN, pulmonary disease, CHF
				[per 1 mg/dL↑ in UA] (LMM)	
2015		- 26 months	2) Time to RRT	2) HR 0.97 (0.93, 1.02)	
				[per 1 mg/dL↑ in UA]	
**Studies showed positive association**					
Iseki^37^	48,177	- Healthy adults	Time to RRT	HR 2.00 (0.90, 4.44) in men	SCr, Age, SBP, DBP, BMI, proteinuria, T-CHO, TG, Hematocrit, glucose
2004		- 6 years		HR 5.77 (2.31, 14.42) in women	
				[for hyperuricemia (UA>7 mg/dL)]	
Yen^32^	591	- Healthy elders	Rapid renal progression (eGFR of ≥ 3 mL/min/1.73m^2^ /y)	OR 1.21 (1.05, 1.39)	SCr, Age, sex, proteinuria, smoking, BMI, BUN, albumin, T-CHO, Hb, WBC, platelet, HTN, DM
2009		- 32.4 months		[per 1 mg/dL↑ in UA]	
		
Chonchol^33^	5,808	- Healthy elders	Rapid renal progression (eGFR> 3 mL/min/1.73 m^2^ /y)	OR 1.49 (1.00, 2.22)	SCr, age, sex, race, weight, SBP, DBP, HTN, medications, diuretics, glucose, allopurinol, lipid, Hb, CRP, albumin, ankle-arm index, carotid IMT, and major ECG abnormalities
2007		- 6.6 years		[Quintiles 5 vs. 1 UA]	
Hsu^34^	177750	- Insured healthy adults	Time to RRT	HR 2.14 (1.65, 2.77)	SCr, older age, DM; education, race, BMI, HTN, proteinuria, glucose, Hb
				[Highest vs. lowest quartile UA]	
2009		- 5,275,957 person-years			
Bellomo^31^	900	- Healthy adults	Rapid renal progression (eGFR> 10 mL/min/1.73m^2^ at 5 years)	HR 1.28 (1.12,1.48)	Age, sex, BMI, glucose, T-CHO, MBP, proteinuria, TG
				[per 1 mg/dL↑ in UA]	
2010		- 59 months			
Kuo^30^	63,758	- Healthy adults	Accelerated eGFR decline (eGFR> 3 mL/min/1.73 m^2^ /y)	HR 1.28 (1.23,1.33)	eGFR, age, sex, DM, HTN, azotemia, T-CHO, TG
				[per 1 mg/dL↑ in UA]	
2011		- 3.0 years			
Dawson^38^	6,984	- Hypertensive patients	Annual eGFR change within the first 5 years	β = -10.7 (-13.6, -7.9) in men	eGFR, age, BMI, prevalent CVD, alcohol, Tobacco,
				β = -12.2 (-15.2, -9.2) in women	
2013		- 5 years		[Highest vs. lowest quartile] (GEE)	
Iseki^39^	16,630	- Healthy adults	eGFR decline over 10 years	eGFR decline: 4.19, 1.91, 2.36, 2.01 ml/min/1.73m2	eGFR, age, sex, HTN, DM
2013		- 10 years		[per 1 mg/dL↑ in UA] in four UA categories (All p<0.05)	
Chang^40^	701	- CKD stage 3–4	1) Rapid renal progression (30% decline in eGFR)	1) OR 1.61(1.27, 2.07)	eGFR, age, sex, DMN, SBP, Hb, Alb, Na, K, P, LDL-C, proteinuria, ACEI/ARB
				[per 1 mg/dL↑ in UA]	
				2) No association	
2015		- 4.5 years	2) Time to RRT	[per 1 mg/dL↑ in UA]	
Uchida^41^	803	- CKD stage 3–4	Time to RRT	HR 2.49 (1.12, 5.52) [for hyperuricemia (UA≥6.5 mg/dL)]	eGFR, Age, sex, DMN, proteinuria
2015		- 4.0 years			
**Studies performed in subpopulation**					
Ficociello^42^ 2010	355	- Type 1 diabetes	Rapid renal progression (loss of GFR_cystatin_ > 3.3% / y)	OR 1.4 (1.1, 1.8)	eGFR(baseline GFR_cystatin)_, sex, HbA1C, proteinuria
		- 4.9 years		[per 1 mg/dL↑ in UA]	
Altemtam^43^ 2012	270	- Type 2 diabetes with CKD stage 3–4	Rapid renal progression (Loss of GFR>2 mL/min/1.73m^2^ /y)	OR 1.16 (1.09, 1.39)	Age, sex, race, HbA1c, SBP, proteinuria, vascular co-morbidities
		- 5.2 years		[per 1 mg/dL↑ in UA]	
Syrjenan^44^ 2000	223	- IgA nephropathy	Rapid renal progression (Scr >120 umol/L in men and >105 umol/L in women) and > 20% elevation from baseline	HR 4.6 (1.1, 19.4) for hyperuricemia (UA>0.45 mmol/l in men and > 0.34mmol/l in women)	Age, sex, HTN, DM, proteinuria, BMI, T-CHO
		- 10 years			
Ohno^45^	748	- IgA nephropathy	the change of CCr	β = 0.143±0.071 for hyperuricemia (UA>7mg/dL) (Linear regression)	HTN, renal pathology
		- 124.4 months (Men)			
2001		- 118.7 months (Women)			
Shi^20^	353	- IgA nephropathy	Rapid renal progression (eGFR decline ≥50%) or RRT or death	HR 2.5 (1.5, 6.1) for hyperuricemia (UA>7 in men, UA>6 mg/dL in women)	eGFR <60 ml/min, HTN, TG, higher Lee’s histological grade, ACEI/ARB
2012		- 5 years			
Meier-Kriesche^50^	1,645	Renal transplant recipients	GFR at 3 year	β = 0.02±0.5	eGFR, Donor type, immunosuppressive treatment arm, ethnicity
		(Symphony study)		[per 1 mg/dL↑ in UA]	
2009		- 3 years		(General linear models)	
Bandukwal^48^	405	Renal transplant recipients	The slope of eGFR	t = 5.59, p = 0.01	Age, newly-onset CVA
2009		6.0 years (Normal UA gourp)		[for Hyperuricemia (UA>7.1 in men and >6.1 mg/dL in women)]	
		7.3 years (Hyperuricemia group)		(Linear regression)	
Haririan^49^ 2012	212	- Renal transplant recipients	1) SCr change at 1^st^ year	1) 1-year serum creatinine:	SCr (mean first 6 month of post transplant), donor and recipient sex, acute rejection during the first year
				β = 0.10 (0.02, 0.18)	
				[per 1 mg/dL↑ in UA]	
				β = 0.25 **(**0.01, 0.49) for hyperuricemia (UA>7 in men, UA>6.5 mg/dL in women)	
		- 68.3 months	2) eGFR change at 1st year	1-year eGFR:	
				β = **-**3.9 (-5.7, -1.5)	
				[per 1 mg/dL↑ in UA]	
				β = **-**7.6 (-13.6, -1.5) for hyperuricemia	
				(Linear regression)	
Kim ^47^	556	- Renal transplant recipients	Serial changes of eGFR	HR 1.454 (1.314, 1.609)	eGFR, Age
2011		- 102.63 months		(per 1 mg/dL↑ in UA)	

**Abbreviations:** HR, hazard ratio; OR, Odds ratio; LMM, Linear mixed model; ACEI, angiotensin converting enzyme inhibitor; ACR, albumin-creatinine ratio; Alb, Albumin; ARB, AT1 receptor antagonist; BMI, Body Mass Index; BUN, blood urea nitrogen; Ccr, creatinine clearance; Chol, cholesterol; CHF, congestive heart failure; CVA, cerebral vascular accident, DM, diabetes mellitus; DKD, diabetic kidney disease; eGFR, estimated glomerular filtration rate; HbA1C, hemoglobin A1c; Hb, Hemoglobin; HCO3, biocarbonate; HTN, hypertension; IHD, ischemic heart disease; IMT, intima-media thickness; MAP, mean arterial pressure; PRD, primary renal disease; SCr, serum creatinine; RRT, renal replacement therapy; T-cho, total cholesterol; TG, triglyceride; WBC, white blood cell.

Superscripted numbers indicate the corresponding reference numbers.

The association between hyperuricemia and the rapid decline in renal function has also been evaluated in subpopulations, including patients with diabetes, IgA nephropathy, and kidney transplantation (Table 4) [20, 43–46]. In 355 patients with type 1 diabetes, a significant positive association was observed between serum UA level and the rapid GFR loss (defined as eGFR decline exceeding 3.3% per year)[43]. In patients with type 2 diabetes who had high normoalbuminuria or microalbuminuria, baseline UA was associated with a faster decline in renal function (defined as loss of eGFR >2 mL/min/1.73m^2^/year) [44]. For IgA nephropathy, three studies showed hyperuricemia was an independent risk factor for progression of IgA nephropathy [20, 45, 46]. Regarding the role of UA in the graft function, the results are heterogeneous [47–51]. In the largest cohort of 1,645 renal transplant recipients, Meier-Kriesche *et al*. found that UA levels at one month after transplantation were not associated with the 3-year graft function [51]. However, other studies suggested UA was associated with rapid loss of graft function and poor graft survival (Table 4) [48–50]. The discrepancy of previous reported findings may be due to a lack of consistency in the definitions of CKD progression, the duration of follow-up, outcome measurements and the covariates adjusted in multivariable models (Table 4). The large inter-study variability in information bias, sample size, and statistical power calls for future large-scale prospective studies.

Although our findings suggest UA might be a modifiable risk factor for the progression of CKD, we did not observe the beneficial effect of allopurinol on CKD progression. One important question is whether the effect of urate-lowering agents can directly abrogate the progression of CKD in humans. According to a recent systemic review including 8 randomized trials, meta-analysis of 5 trials (346 participants) using eGFR decline as the study outcome showed no difference between allopurinol and placebo group while the other 3 trials (156 participants) using change in serum creatinine as the end-point found patients received allopurinol therapy had significantly lower serum creatinine concentrations comparing to placebo group[17]. The discrepancy may be due to only a small number of single-center trials available and the inconsistency in outcome measurements [17]. One caveat of allopurinol is serious side effects, such as severe allergic reaction, may lead to higher morbidity even mortality in CKD patients before the potential beneficial effects on kidney function are observed [52]. Whether the newer urate-lowering agents with fewer side effects, such as febuxostat [53], may have any benefit in controlling the progression of CKD requires further research.

Several potential mechanisms that UA may hasten the progression of renal function have been proposed. The precipitation of urate in renal tubules may cause “uric acid nephropathy” [54]. Besides, the crystallization of the UA in the renal medullary interstitium can induce secretion of IL-1β, which may recruit more inflammatory cells and cause chronic interstitial inflammation and fibrosis [55–59]. Hyperuricemia may also induce proliferation of vascular smooth muscle cells and increase COX-2 expression and renal renin, leading to arteriopathy and hypertension–which may further aggravate kidney function[55].

Strengths of this study include the longitudinal methodology to avoid issues of cross-sectional design such as potential reverse causality and the laboratory’s consistency in the measurement of serum UA and creatinine throughout the duration of follow-up. From our study, uric acid is not just solely as a marker, but a true mediator for CKD. Moreover, compared to previous studies using relatively small sample size (<100) to evaluate the role of urate-lowering agents in the CKD progression in a shorter period of follow-up (less than 2 years)[17], we estimated the effect of UA and allopurinol use on renal function in a larger sample with longer follow-up time. The study also had some limitations. First, we only evaluated the effect of baseline UA level on the progression of kidney function. Future studies should focus on the association between longitudinal trajectories of both UA and eGFR. Second, residual confounding could not be completely excluded since this study did not have information on other nephrotoxic or renoprotective agents (e.g., aminoglycosides or contrast) and adherence of urate-lowering agents. Independent non-differential misclassification of some proposed confounders may also lead to imperfect adjustment for confounding. For instance, the blood pressure was only measured once at baseline clinic visit. Although the use of multiple criteria to define hypertension should have greatly minimized the misclassification error, we acknowledge that our findings should be interpreted with caution and future research replicating these findings in large clinical samples is warranted. Third, the usage of diuretics was not obtained at the time of data collection. Although diuretics use may associate with increased serum uric acid level, the association between diuretic use and eGFR decline is controversial [26]. Therefore, our study results should be robust with and without adjusting for the usage of diuretics [60]. Fourth, the etiologies of hyperuricemia were not determined systematically in this study. Similar to other clinical studies investigating the role of UA in the renal function, such information is rarely available. Whether our findings remain consistent in patients with different hyperuricemia mechanisms (e.g., primary vs. secondary; over-production vs. hypo-excretion) requires further research.

## Conclusions

Our study showed a higher UA level was significantly associated with a greater decline in renal function and a higher risk of progressing to kidney failure in a Chinese population. The influence of hyperuricemia on renal function decline and risk of renal failure was greater in subjects without proteinuria than in those with proteinuria. In addition, allopurinol did not have a benefit in mitigating CKD progression. Our findings support that hyperuricemia as a potential modifiable risk factor for CKD progression, particularly in patients without proteinuria.

## Supporting Information

S1 Fig The flow chart of our retrospective cohort established based on electronic medical records (EMR). (TIF)

S1 Table Variables associated with changes in eGFR among the cohort using linear mixed model. (DOCX)

S2 Table Variables associated with CKD progression to kidney failure (eGFR<15ml/min). (DOCX)
