# Supplementary figures and images for: Serum Uric Acid and Progression of Kidney Disease: A Longitudinal Analysis and Mini-Review
Source: PLoS One. 2017 Jan 20;12(1):e0170393. doi: 10.1371/journal.pone.0170393 (PMC5249245; doi:10.1371/journal.pone.0170393)

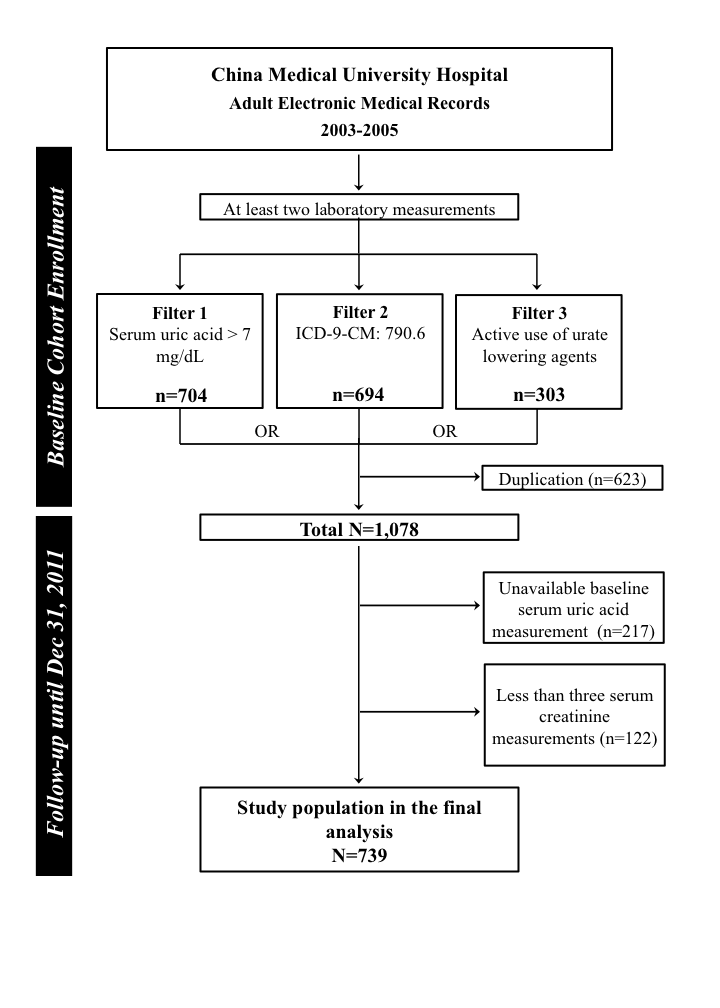

Supplement: S1 Fig — (TIF) [file pone.0170393.s001.tif]
