# Supplementary material for: Serum Uric Acid and Progression of Kidney Disease: A Longitudinal Analysis and Mini-Review
Source: PLoS One. 2017 Jan 20;12(1):e0170393. doi: 10.1371/journal.pone.0170393 (PMC5249245; doi:10.1371/journal.pone.0170393)
Supplement: S1 Table — (DOCX) [file pone.0170393.s002.docx]

**S1 Table.** Variables associated with changes in eGFR among the cohort using linear mixed model

|  | β | S.E. | *p* |
| --- | --- | --- | --- |
| Uric acid (mg/dL) |  |  |  |
| UA < 6 | Reference |  |  |
| UA 6-8 | -11.2 | 2.4 | <0.001 |
| UA 8-10 | -12.6 | 2.4 | <0.001 |
| UA > 10 | -13.1 | 2.8 | <0.001 |
| Age at exam (year) | -0.6 | 0.1 | <0.001 |
| Male | 1.9 | 1.8 | 0.3 |
| BMI | -0.08 | 0.19 | 0.7 |
| DM | -1.7 | 1.7 | 0.3 |
| CAD | 0.5 | 2.1 | 0.8 |
| SBP (mmHg) | -0.02 | 0.03 | 0.7 |
| Allopurinol | -9.8 | 1.8 | <0.001 |
| Baseline creatinine (mg/dL) | -7.4 | 0.4 | <0.001 |
| Proteinuria (Yes/no) | -9.1 | 1.7 | <0.001 |
| ACEI | -6.1 | 1.8 | 0.001 |

*S.E.: standard error
