# Supplementary material for: Serum Uric Acid and Progression of Kidney Disease: A Longitudinal Analysis and Mini-Review
Source: PLoS One. 2017 Jan 20;12(1):e0170393. doi: 10.1371/journal.pone.0170393 (PMC5249245; doi:10.1371/journal.pone.0170393)
Supplement: S2 Table — (DOCX) [file pone.0170393.s003.docx]

**S2 Table.** Variables associated with CKD progression to kidney failure (eGFR<15ml/min)

|  | HR | 95% C.I. | *p* |
| --- | --- | --- | --- |
| Uric acid | 1.12 | 1.05-1.20 | 0.001 |
| Age at exam | 1.03 | 1.01-1.04 | <0.001 |
| Sex | 0.77 | 0.53-1.12 | 0.17 |
| BMI | 0.96 | 0.92-1.01 | 0.09 |
| DM | 1.34 | 0.92-1.96 | 0.13 |
| CAD | 0.41 | 0.30-0.83 | 0.007 |
| SBP | 1.01 | 1.00-1.02 | 0.001 |
| proteinuria | 4.02 | 2.57-6.29 | <0.001 |
| Allopurinol | 1.11 | 0.75-1.65 | 0.59 |
| ACEI | 0.62 | 0.41-0.94 | 0.023 |
| Baseline creatinine | 1.18 | 1.13-1.24 | <0.001 |
